# Aftermath: A Word to Appalachia Following Hurricane Helene

**DOI:** 10.13023/jah.0603.01

**Published:** 2024-10-01

**Authors:** Emily Wilson

**Affiliations:** East Tennessee State University

**Keywords:** Appalachia, public health

## Abstract

On September 27, 2024, Hurricane Helene caused massive and catastrophic flash flooding in the lower Appalachian Region, leveling towns and forever altering much of the landscape. This is an open letter to the people of Appalachia who were affected by the disaster that blindsided our region.

Hours after I was offered the position of managing editor with the *Journal*, I went on a long drive into Johnson County with my family. My heart was immeasurably full; in the cupped hands of the Appalachian Mountains, I was soulfully renewed with the knowledge that I would be exactly where I needed to be – involved in my community, giving my skill and knowledge to the area I have (and always will) call home. This complete tranquility, this settledness of my heart, reflected vibrantly in the richness of the landscape just beyond the car window: blurred greens, rolling hills, idyllic and familiar. It is a blessing, truly, to live so close to the foothills of mountains that have quietly observed both history and prehistory. I returned home feeling the way one feels after a particularly satisfying church service. Only three short months later, Hurricane Helene would ravage Appalachia.

There is something remarkably special about these mountains. More than their beauty, their sublimity, there is the spirit of resilience – not only of the mountains themselves, but the people within them. “Mountain folk,” we’re called. Through wildfires, global misunderstanding, and the heartache of environmental destruction, we persist. Nothing, though, has quite highlighted this hardiness like the recent devastation experienced in this region by the floodwaters of Helene. Lives were lost; entire towns were wiped from the map; houses floated downriver. My heart has broken over and over again with each new photo, each news broadcast, each story I hear from those directly affected. The land, the people I have loved and known to be an intrinsic, core piece of me, have seen devastation previously unimaginable.

The grief that I and all of my fellow Appalachians feel right now is real. It is tangible; it is evidenced in the indelibly altered terrains of Asheville, Chimney Rock, Erwin, Unicoi, Greeneville, Roan Mountain – the list goes on. This grief echoes and rebounds; we lost more than land, more than history, and each and every one of us feels the loss of those who are no longer with us. But we must remember that grief is the shadow of love, and that love does not die until we allow it. In the face of devastation, we must do what mountain folk do best: remember, persist, remain.

We at the *Journal of Appalachian Health* encourage those affected by this tragedy to write about their experiences and share them with us – be they medical, structural, or personal. A sorrow shared is a sorrow halved, and each voice has its place within our pages.

